# Low numbers of blood and salivary natural killer cells are associated with a better response to belimumab in primary Sjögren’s syndrome: results of the BELISS study

**DOI:** 10.1186/s13075-015-0750-y

**Published:** 2015-09-04

**Authors:** Raphaèle Seror, Gaétane Nocturne, Thierry Lazure, Houria Hendel-Chavez, Frédéric Desmoulins, Rakiba Belkhir, Philippe Ravaud, Mohcine Benbijja, Vichnou Poirier-Colame, Yacine Taoufik, Xavier Mariette

**Affiliations:** Université Paris-Sud, Center of Research on Immunology of Viral and Autoimmune diseases (IMVA), INSERM U1184, Le Kremlin Bicêtre, France; Université Paris-Sud, Assistance Publique-Hôpitaux de Paris (AP-HP), Service de Rhumatologie, Hôpitaux Universitaires Paris-Sud, Hôpital Bicêtre, 78 rue du Général Leclerc, 94275 Le Kremlin Bicêtre, France; Université Paris-Sud, Assistance Publique-Hôpitaux de Paris (AP-HP), Service d’anatomopathologie, Hôpitaux Universitaires Paris-Sud, Le Kremlin Bicêtre, France; Université Paris-Sud, Assistance Publique-Hôpitaux de Paris (AP-HP), Laboratoire d’immunologie, Hôpitaux Universitaires Paris-Sud, Le Kremlin Bicêtre, France; Université Paris Descartes, AP-HP, Hôtel-Dieu Hospital, Centre de Recherche Épidémiologie et Statistique Sorbonne Paris Cité (CRESS-UMR1153), Paris, France; Institut de Cancérologie Gustave Roussy Cancer Campus (GRCC), INSERM U1015, GRCC, 475 rur Edouard Vaillant, 94805 Villejuif, France

## Abstract

**Introduction:**

In this study, we sought to address changes in blood lymphocyte subpopulations and labial salivary gland (LSG) inflammation after belimumab treatment in patients with primary Sjögren’s syndrome (pSS) and to identify predictors of response to treatment.

**Methods:**

Sequential blood lymphocyte subsets and LSG biopsies were analysed between week 0 (W0) and W28 in 15 patients with pSS treated with belimumab. Systemic response to treatment was defined as a decrease in the European League Against Rheumatism Sjögren’s Syndrome Disease Activity Index score of ≥3 points at W28.

**Results:**

After belimumab, we observed a decrease in blood B lymphocytes primarily involving CD27-negative/immunoglobulin D–positive naïve B cells (*p*=0.008). Lymphocytic sialadenitis (focus score >1) that was present in 12 patients (80.0 %) before belimumab treatment became negative in 5 of them after treatment (*p*=0.03). The median (interquartile range) LSG B-cell/T-cell ratio decreased from 0.58 (0.5–0.67) to 0.50 (0.5–0.5) (*p*=0.06). B-cell activating factor (BAFF) staining was detected in 11 (78.6 %) of 14 patients before belimumab treatment compared with 7 (50.0 %) of 14 after belimumab therapy (*p*=0.10). The median percentage of BAFF-positive cells in foci significantly decreased from 27.5 % (10–40) to 5 % (0–20) (*p*=0.03). A systemic response was achieved in six patients (40 %). The only predictor of response was the presence of a low number of natural killer (NK) cells, both in blood (8.5 % [7–10] vs 11 % [9–21]; *p*=0.04) and in LSG (20.6/mm^3^ [20.0–21.4] vs 30.0/mm^3^ [25.0–100.0], *p*=0.003). Serum BAFF levels did not influence response to treatment.

**Conclusions:**

Low blood and salivary NK cell numbers are associated with a better response to belimumab. This suggests that two distinct subsets of pSS may exist: one with a predominant type I interferon (IFN)–BAFF–B-cell axis, representing good responders to belimumab; and one with a predominant type II IFN–NK cell axis, representing non-responders.

**Trial registration:**

ClinicalTrials.gov identifier: NCT01160666. Registered 9 July 2010.

## Introduction

Primary Sjögren’s syndrome (pSS) is a systemic autoimmune disease that is characterised by mouth and eye dryness as well as fatigue and pain. Systemic manifestations occur in 20–40 % of patients [1, 2]. B lymphocytes play a crucial role in the pathogenesis of the disease in their capacity to secrete autoantibodies and cytokines or to present autoantigens.

Increased expression of the cytokine B-cell activating factor (BAFF), also called *B-lymphocyte stimulator* [3], is involved in the pathogenic mechanism of B-cell activation. BAFF plays a crucial role in B-cell maturation, plasma cell survival, antibody response promotion and immunoglobulin (Ig) class-switching recombination [3, 4]. Its involvement in the pathogenesis of autoimmune diseases and lymphomagenesis is demonstrated by BAFF-transgenic mice that have autoimmune diseases mimicking systemic lupus erythematosus (SLE) and pSS and a rate of B-cell lymphoma twice that of control mice [4]. Patients with SLE and pSS have elevated serum levels of BAFF [5, 6], and serum levels of BAFF correlate with autoantibody levels [6–8] and have been found to be associated with pSS-associated lymphoproliferative complications [9, 10].

Therefore, targeting B-cell activation and BAFF in the setting of pSS seems appealing. Belimumab is the first marketed anti-BAFF monoclonal antibody. It was recently approved for treatment of SLE on the basis of two phase III studies that showed positive results [11, 12]. Because pathophysiological studies also suggested an involvement of BAFF in the pathogenesis of pSS, we conducted the first open-label proof-of-concept study to evaluate the efficacy and safety of belimumab in pSS and found promising clinical results, including a decrease in disease activity as assessed using the European League Against Rheumatism Sjögren’s Syndrome Disease Activity Index (ESSDAI) and in patients’ symptoms as assessed using the European League Against Rheumatism Sjögren’s Syndrome Patient Reported Index (ESSPRI).

As a part of the present trial, we addressed the changes in labial salivary gland (LSG) inflammation and serum lymphocyte pattern after belimumab therapy and identified predictors of response to treatment. We tried to find patient patterns corresponding to probable involved pathogenic pathways to make a first step toward personalised medicine in this polymorphic disease.

## Methods

### Patients

The Efficacy and Safety of Belimumab in Subjects with Primary Sjögren’s Syndrome (BELISS) trial included patients in two identical studies conducted at the same time in two European centres, one in Paris, France, and one in Udine, Italy (ClinicalTrials.gov registration numbers NCT01160666 and NCT01008982). Patients included fulfilled the American-European Consensus Group criteria for pSS [13], were positive for anti-Sjögren’s syndrome antigen A or anti–Sjögren’s syndrome antigen B antibodies and had, at the time of inclusion, at least one of the following three characteristics: systemic complications or persistent salivary gland enlargement, early disease (≤5 years from the beginning of symptoms) and/or presence of at least one biomarker of B-cell activation (increase in IgG level or free light chains or β_2_-microglobulinemia, decrease in complement component 4 [C4] level, presence of cryoglobulinemia or monoclonal component). Other inclusion and exclusion criteria are reported elsewhere [14]. The patients received belimumab 10 mg/kg at week 0 (W0), W2 and W4 and then every 4 weeks to W24. Patients who responded to treatment at W28 were continued with belimumab monthly through W48, with a final evaluation scheduled at W52 (4 weeks after the last dose).

The present study is part of the BELISS trial and included analysis of changes in histological and serum lymphocyte patterns between W0 and W28 in the 15 patients at the French centre.

### Definitions of response to treatment

Response to treatment was defined at W28 according to the composite primary endpoint, defined as follows: improvement in two of the following five parameters at W28: ≥30 % reduction in dryness score on a visual analogue scale (VAS), ≥30 % reduction in fatigue score on a VAS, ≥30 % reduction in musculoskeletal pain score on a VAS, ≥30 % reduction in systemic activity score on a VAS assessed by the physician, and/or ≥25 % reduction in serum levels of any of several B-cell activation biomarkers (free light chains of Ig, β_2_-microglobulin, monoclonal component, cryoglobulin, IgG) or ≥25 % increase in C4 level.

Systemic response was also assessed at W28 and was defined as a decrease of ≥3 points in ESSDAI score [15] in accordance with its minimal clinically important improvement [16]. Analyses of factors associated with response to treatment were based primarily on systemic response, which we considered the most robust way to define relevant improvement.

### BAFF assessment

BAFF was measured at baseline, before the first belimumab dose, using an enzyme-linked immunosorbent assay (Quantikine kit; R&D Systems, Minneapolis, MN, USA).

### Flow cytometry

Subtype lymphocyte counts for T, B and natural killer (NK) cells were obtained by flow cytometry at W0, W4, W12 and W28. The results are expressed in absolute value (number of cells per cubic millimetre of blood). A blood sample of 200 μl was used for phenotyping subsets of CD19^+^ with the following antibodies CD19 peridinin-chlorophyll protein (PerCP, clone 4GT, catalogue number 345778; BD Biosciences, San Jose, CA, USA), IgD fluorescein isothiocyanate (catalogue number H15501; Invitrogen, Carlsbad, CA, USA) and CD27 allophycocyanin (clone L128, catalogue number 337169; BD Biosciences). After staining, the blood sample was fixed (Lyse/fix solution; BD Biosciences) and cells were acquired on a BD FACSCalibur flow cytometer (BD Biosciences).

Among B cells, the following subtypes were analysed: naïve (CD27^−^IgD^+^) B cells, memory (CD27^+^) B cells and their subtypes (switched [CD27^+^IgD^−^] and unswitched [CD27^+^IgD^+^]).

### Histological assessment

Minor LSG biopsy samples were obtained at W0 and W28. Samples of LSGs were fixed in alcohol, acetic acid and formaldehyde solution and embedded in paraffin wax for histological study. All samples were analysed by the same pathologist (TL).

The following parameters were analysed: the focus score, the B-cell/T-cell ratio in the foci, BAFF expression in the foci and the NK infiltrate inside and outside the foci. The focus score was measured after haematoxylin eosin staining and was expressed as the number of foci (50 lymphocytes) per 4 mm^2^. The focus score was considered abnormal if it was ≥1. B cells and T cells were identified after CD20 (clone L26; DakoCytomation, Glostrup, Denmark) and CD3 (clone F.7.2.38; DakoCytomation) staining, respectively. The B-cell/T-cell ratio was estimated in foci and expressed as the proportion of B cells to T cells. BAFF expression was measured after incubation with a rat anti-human BAFF antibody called Buffy-2 (Enzo Life Sciences, Villeurbanne, France) and expressed as the proportion of BAFF-expressing cells inside the foci, regardless to their origin (B or T cells). NK cell infiltration was analysed after incubation with mouse anti-human NKP46/NCR1 antibody (R&D Systems) inside and around the foci and was expressed as the number of NK cells per square millimetre as previously described [17].

### Ethics

The study protocol was approved by the French ethics committee protection of humans in biomedical research and the French agency for the safety of medicine and health products. The study was conducted according to the current regulations of the International Conference on Harmonisation guidelines and the principles of the Declaration of Helsinki. All patients provided written informed consent before participating in any protocol-specific procedures.

### Statistical analyses

Categorical variables are described with numbers (percentages). Quantitative variables are described with the median (interquartile range).

To analyse changes between W0 and W28, values were compared using Wilcoxon’s signed-rank test for quantitative variables and McNemar’s test for categorical variables.

To identify predictive factors of response to treatment, baseline lymphocyte counts and histological parameters were compared between responders and non-responders, using a Kruskal-Wallis test for quantitative variables and χ^2^ or Fisher’s exact test for categorical variables.

For all statistical analyses, a *p* value <0.05 was considered statistically significant. Data analysis was carried out with SAS 9.3 for Windows software (SAS Institute, Cary, NC, USA).

## Results

### Patients

All 15 patients were women. Their median age was 44 (36.5–63.5) years. Their median disease duration was 1 (0.5–6.5) year, and their median duration of symptoms was 6.5 (4–12.5) years. Their characteristics are reported in Table 1.

**Table 1:** Baseline characteristics of patients

	All patients (N = 15)
Age, yr, median [IQR]	44 [36.5–63.5]
Female sex (%)	15 (100.0 %)
Disease duration, yr, median [IQR]	1 [0.5–6.5]
Whole unstimulated salivary flow (<0.1 ml/min)	12 (80 %)
Schirmer’s test ≤5 mm	10 (66.7 %)
Focus score ≥1	12 (80 %)
Baseline focus score, median [IQR]	1.6 [1–2.4]
Anti-SSA antibodies	14 (93.3 %)
Anti-SSB antibodies	10 (66.7 %)
Current background medication	
Corticosteroids	4 (26.7 %)
Hydroxychloroquine	5 (33.3 %)
Methotrexate	0 (0 %)
Reason for inclusion	
Systemic complications	11 (73.3 %)
Recent-onset disease	4 (26.7 %)
Increase in B-cell biomarker values	11 (73.3 %)
ESSDAI score (0–123), median [IQR]	7 [3–11]
ESSPRI score (0–10), median [IQR]	6.33 [6–7.33]

*Anti-SSA* anti-Sjögren’s syndrome antigen A antibodies, *anti-SSB* anti-Sjögren’s syndrome antigen B antibodies, *ESSDAI* European League Against Rheumatism Sjögren’s Syndrome Disease Activity Index, *ESSPRI* European League Against Rheumatism Sjögren’s Syndrome Patient Reported Index, *IQR* Interquartile range

Among the 15 patients, 8 had salivary gland swelling at the time of inclusion and 11 had other systemic complications, such as articular involvement (n=6), pulmonary involvement (n=2), muscular involvement (n=1), peripheral neuropathy (n=1), renal tubular acidosis (n=1) and autoimmune thrombocytopenia (n=1).

### Belimumab induced incomplete B-cell depletion mainly linked to a dramatic decrease of naïve B cells

The available T, B and NK cell fluorescence-activated cell-sorting analysis data allowed the evaluation of 13 patients at baseline and comparisons between W0 and W28 in 11 patients. Regarding B-cell subtypes, data were available at baseline for nine patients and for comparison between W0 and W28 for eight patients.

**Fig. 1:**
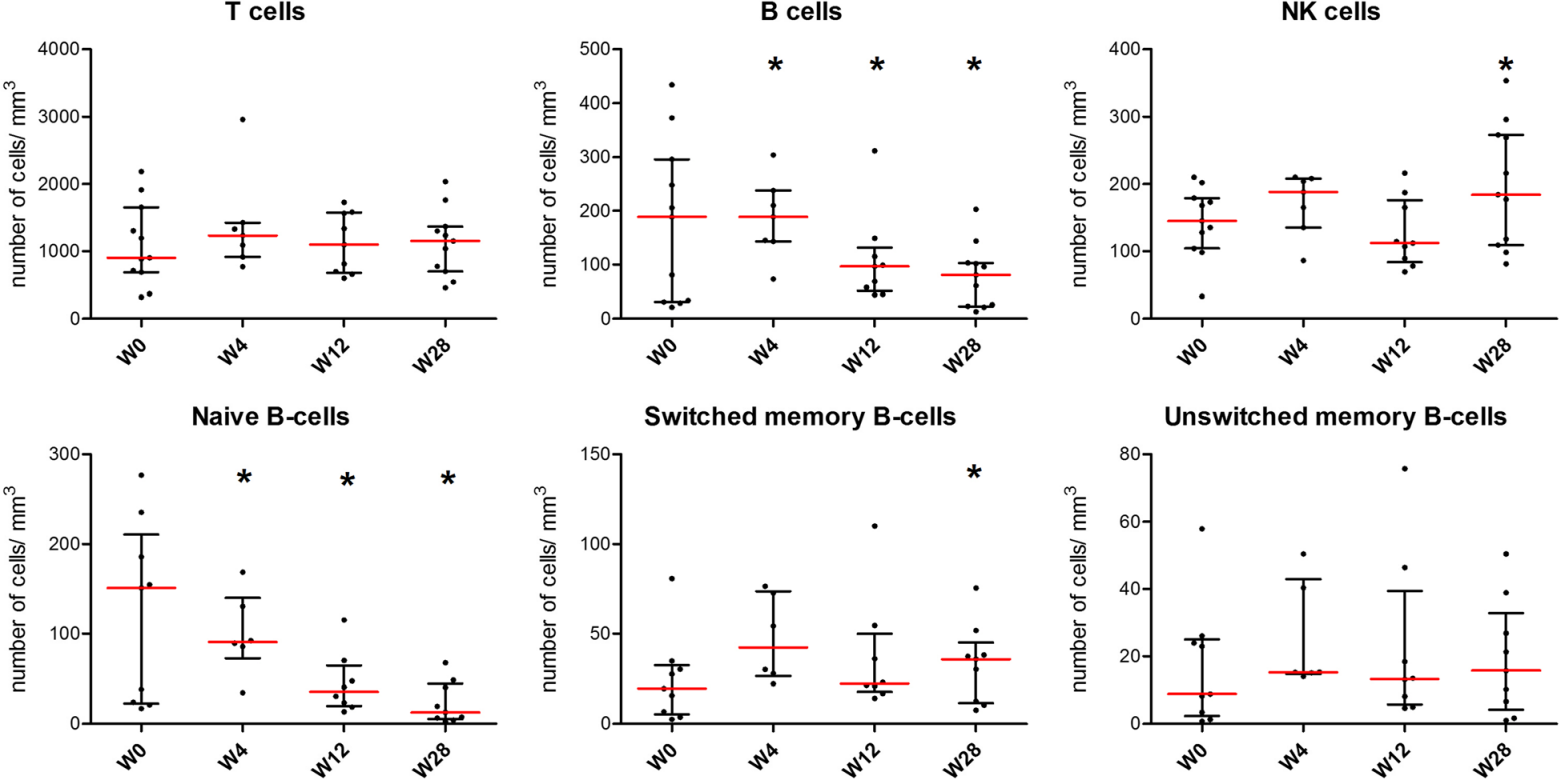
Changes in T, B and natural killer (NK) cell counts and B-cell subtypes after belimumab therapy. *p<0.05. *W* week

After belimumab treatment, the median total lymphocyte count (1346 [895–2069] at W0 vs 1471 [905–1847] at W28; n=11), T cell count (902 [689–1655] at W0 vs 1152 [697–1367] at W28; n=11), CD4 (690 [471–890] at W0 vs 724 [438–923] at W28; n=11) or CD8 T cells (336 [135–541] at W0 vs 388 [185–544] at W28; n=11) did not change significantly (Fig. 1a).

By contrast, there was a significant increase in the number of NK cells (145 [104–179] at W0 vs 184 [109–273] at W28, n=11; *p*=0.032) (Fig. 1c). This number did not differ according to the presence of systemic complications or glandular involvement.

Conversely, we found a decrease in B lymphocytes (CD19^+^). This decrease was observed as soon as W4 (i.e., the first evaluation after belimumab initiation) and persisted during the whole treatment duration (189 [31–296] W0 vs 81 [23–103] to W28, n=11; *p*=0.01) (Fig. 1b).

**Fig. 2:**
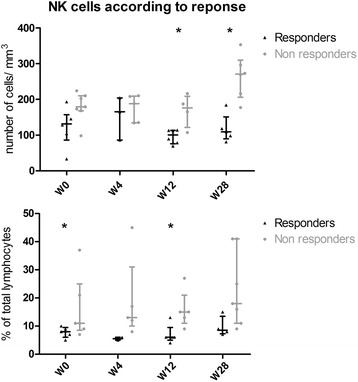
Changes in natural killer (NK) cell count and proportion in responders and non-responders. Number (*upper plot*) and percentage (*lower plot*) of NK cells in responders and non-responders are shown. *p<0.05

This decrease primarily involved CD27^−^IgD^+^ naïve B cells (151 [24–186] at W0 vs 10 [6–40] at W28, n=9; *p*=0.008). There was no significant change in the total number of CD27^+^ memory B cells (39.5 [10.1–53.2] at W0 vs 47.7 [19.1–64.9] at W28, n=9; *p*=0.074) or CD27^+^IgD^+^ unswitched memory B cells (8.8 [3.4–28.9] at W0 vs 15.7 [6.6–28.8] at W28, n=9; *p*=0.36), but there was a slight increase in the number of CD27^+^IgD^−^ switched memory B cells at W28 (19.6 [6.7–30.3] at W0 vs 35.8 [12.5–38.2] at W28; *p*=0.019) (Fig. 2).

**Fig. 3:**
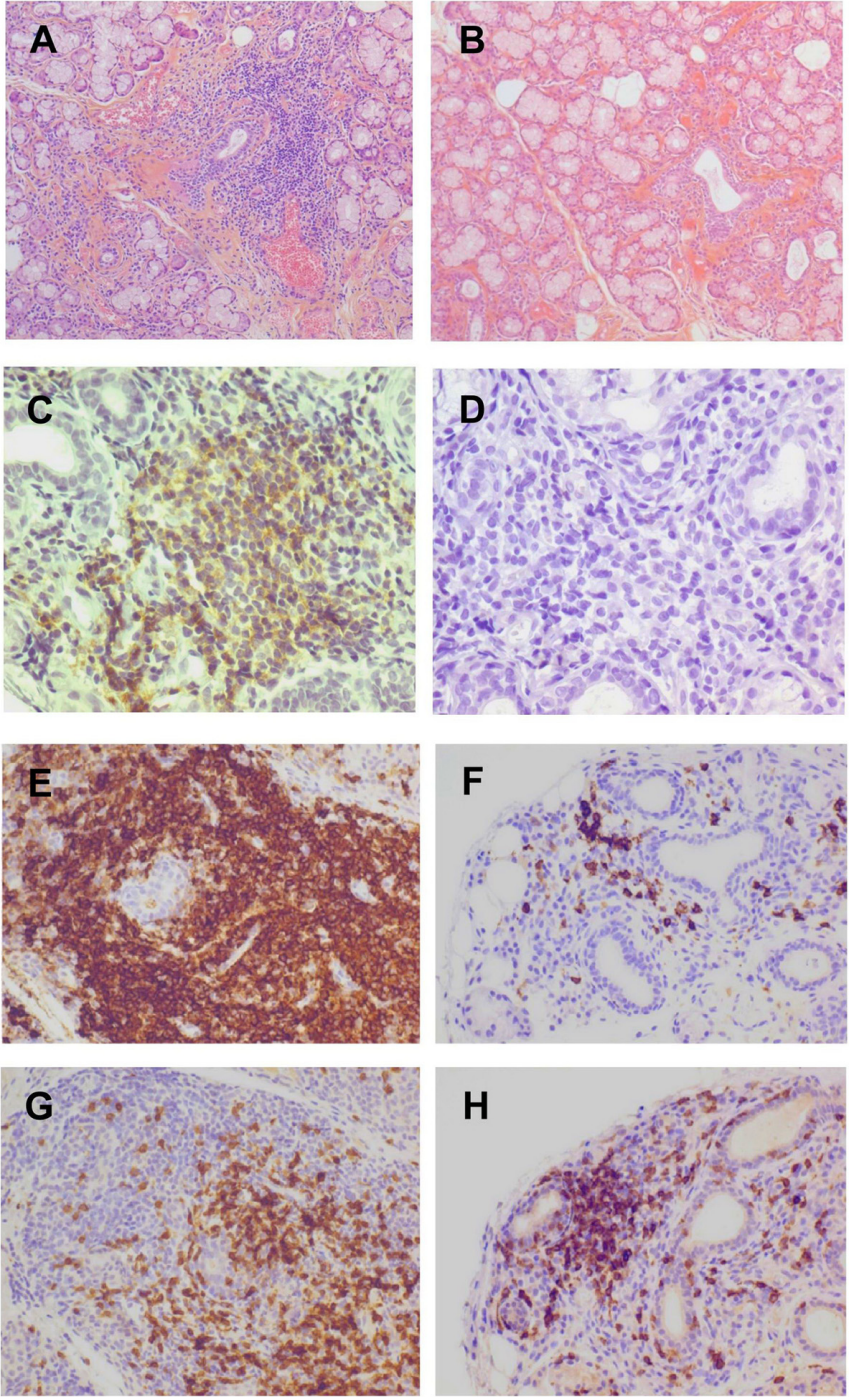
Changes in histological pattern of salivary glands after belimumab therapy. Regression of lymphocytic infiltration after belimumab therapy (**a** before and **b** after; hematein-eosin-saffron stain; original magnification, ×125), major decrease of B-cell activating factor expression (**c** before and **d** after; Buffy-2 immunohistochemical stain, original magnification, ×320.), dramatic decrease of B-cell infiltration (**e** before and **f** after; CD20 immunohistochemical stain; original magnification, ×250), with slight decrease or stability of T-cell infiltration (**g** before and **h** after; CD3 immunohistochemical stain; original magnification, ×250), resulting in a trend for a decrease in B-cell/T-cell ratio

### Belimumab therapy induces decrease of BAFF expression and B-cell/T-cell ratio in salivary glands

Minor LSG samples were obtained for all 15 patients at both W0 and W28. All samples were analysed for estimating focus and Chisholm scores, but only data of 14 patients were analysed for specific staining Fig. 3.

At inclusion, lymphocytic sialadenitis with a focus score >1 was observed in 12 (80 %) of 15 patients, 5 of whom had a negative test at W28 (*p*=0.03). Overall, the median focus score decreased from 1.6 (1–2.4) to 0.5 (0–3) (*p*=0.39) and Chisholm score decreased from 4 (3–4) to 2 (1–4) (*p*=0.007).

The median B-cell/T-cell ratio decreased from 0.58 (0.5–0.67) to 0.50 (0.5–0.5) (*p*=0.06). The presence of BAFF staining was detected in 11 (78.6 %) of 14 patients before belimumab treatment and in only 7 (50.0 %) of 14 after belimumab therapy (*p*=0.10). The median percentage of BAFF-positive cells in foci significantly decreased from 27.5 % (10–40) to 5 % (0–20) after belimumab therapy (*p*=0.03). NKp46 staining revealed that NK cell infiltration was located predominantly in interstitia rather than in foci (24.7 [21.4–38.9] vs 8.2 NKp46^+^ cells/mm^2^ [4.2–10.0]; *p*=0.0003) and did not change after belimumab treatment. Of note, we observed a correlation, also found in one of our previous works, between the grading of focus score and the number of NKp46^+^ cells outside the inflammatory foci (*r*^2^=0.425; *p*<0.0001).

### Low numbers of blood and salivary natural killer cells are associated with a better response to belimumab

At W28, 6 (40 %) of the 15 patients were responders according to the systemic definition (ESSDAI decrease of ≥3 points) and 7 (53 %) achieved the composite primary endpoint, including the 6 systemic responders, showing a high concordance between these 2 criteria. The following analyses were based on systemic response.

Among the initial lymphocyte subpopulations, the only parameters associated with systemic response to belimumab were the percentage and number of NK cells (8.5 % [7–10] in responders vs 11 % [9–21] in non-responders, *p*=0.04; and 131 [104–145] in responders vs 179 cells/mm^3^ [168–210] in non-responders, *p*=0.06). This was further confirmed at W12 (*p*=0.02) and W28 (*p*=0.04), when lower NK cell counts were observed in responders than in non-responders.

Also, change in NK cell absolute counts between W0 and W28 significantly differed between responders and non-responders. Even if they started with a higher number of NK cells, non-responder patients showed a further increase of NK cells compared with relative stability in responders (+108 NK/mm^3^ [67–118] vs −23/mm^3^ [−26, +56]; *p*=0.017).

Neither baseline total nor B-cell subset cell counts, nor their variations with treatment, were associated with response to belimumab. Baseline serum BAFF levels also did not differ between responders and non-responders (790.3 pg/ml [692.1–927.4] vs 885.0 pg/ml [721.9–1100.5]; *p*=0.79).

Regarding histological findings, the only parameter associated with systemic response to belimumab was the importance of NK infiltrate in the periphery of the foci, which was lower in responders than in non-responders (median number of NK cells 20.6 [20.0–21.4] vs 30 NKp46^+^ cells/mm^2^ [25.0–100.0]; *p*=0.003).

Neither the percentage of BAFF-positive cells nor B-cell/T-cell ratio was associated with the response to belimumab.

## Discussion

In the present study, we assessed blood and LSG lymphocyte patterns before and after belimumab treatment in patients with pSS. Belimumab treatment was associated with significant and early incomplete B-cell depletion in peripheral blood involving the CD27^−^IgD^+^ naïve B-cell subset. Analyses of LSG biopsies demonstrated a decrease in lymphocytic infiltration as assessed by focus and Chisholm scores. A significant decrease in the percentage of BAFF-positive cells within the foci was also observed. The only predictor of response to belimumab was a low count of blood and salivary NK cells.

Our study has two main limitations. First, it is an open study with no control arm for comparison. Second, the number of patients is small. Nevertheless, even in a small group of patients, we found interesting, significant results that reinforce their robustness and demonstrate that studying a small, well-phenotyped group of patients can be very informative.

The findings of the peripheral blood mononuclear cells are in line with what is known about the effect of belimumab therapy in patients with SLE. It is now established that belimumab leads to a decrease in naïve B cells and to stability or an early increase in memory B cells [18, 19]. We confirmed this interesting observation of an increase in memory B cells after belimumab treatment. We hypothesised that the magnitude of this increase in blood could reflect the importance of memory B-cell infiltration in the tissue as previously described [20]. Indeed, the association of BAFF and chemokine (C-X-C motif) ligand 13 has been shown to be necessary for attracting memory B cells within germinal centres [21]. However, with the limitation of a small number of patients, we did not find any correlation between the magnitude of this increase and the response to treatment. To our knowledge, the monitoring of blood NK cells has not been studied in SLE. In this study, we found an effect of belimumab on NK cells whose absolute count increased with treatment.

We demonstrate for the first time that belimumab may induce changes in LSG infiltrate: decreases of lymphocytic infiltrate, Chisholm score and BAFF-expressing cells in the foci as well as a trend for a decreased B-cell/T-cell ratio. The decrease of BAFF-expressing cells is of particular interest but must be interpreted cautiously. It has been demonstrated in pSS that BAFF is secreted not only by monocytes and/or macrophages and dendritic cells but also by T and B cells and by salivary epithelial cells [22, 23]. However, the lowest number of BAFF-expressing cells could be only the consequence of the decrease of B cells expressing the BAFF receptor, with BAFF passively bound on its receptor and detectable by the anti-BAFF antibody.

The most interesting finding of the study is that, compared with non-responders, patients who responded to belimumab had a lower baseline number of NK cells, both in blood and in LSG at the periphery of the foci. We have previously shown that NK cells were present mainly in the salivary glands in this location and could play a role in pSS [17]. We have hypothesised that these NK cells could participate in the increased interferon (IFN)-γ expression seen in a subset of patients with pSS. Indeed, the IFN signature observed in this disease may be divided in patients harbouring a restricted type I signature, a restricted type II signature or both [24]. On the one hand, type I IFN and B-cell activation could be linked, as supported by studies showing that IFN-α can induce BAFF [23, 25–27] and association between the type I IFN signature and B-cell biomarkers [28]. On the other hand, recent studies have highlighted the role of the interleukin-12–IFN-γ axis in pSS pathogenesis [29]. Therefore, patients with a high baseline number of NK cells in the blood or in the glands might correspond to this second subset of patients having a high type II IFN signature, suggesting a disease not predominantly driven by B cells and thus less responsive to belimumab. Conversely, patients with low blood NK cell count or salivary gland infiltration might be more dependent on the type I IFN–B cell axis and may be good candidates for B-cell–targeted therapy.

Interestingly, the lower NK cell count observed in responders compared with non-responders persisted throughout the study at W12 and W28, suggesting that this difference in NK cell numbers might really be the mirror of two types of pathogenesis in patients with pSS.

## Conclusions

We found very interesting results suggesting an effect of belimumab on the glandular lymphoid infiltrate. One of the main results is the identification of the NK cells as an interesting predictive factor of the efficacy of belimumab, suggesting that two types of disease can be hypothesised in patients with pSS, the first one being NK cell– and type II IFN–driven and not responsive to B-cell–targeted therapy and the other being more B-cell–driven and more responsive to belimumab therapy. If the effectiveness of belimumab is confirmed in patients with pSS in randomised controlled studies, our findings could represent a first step toward personalised medicine for patients with pSS.

## Abbreviations

Anti-SSA: anti-Sjögren’s syndrome antigen A antibodies
anti-SSB: anti-Sjögren’s syndrome antigen B antibodies
BAFF: B-cell activating factor
BELISS trial: Efficacy and Safety of Belimumab in Subjects With Primary Sjögren’s Syndrome
C4: Complement component 4
ESSDAI: European League Against Rheumatism Sjögren’s Syndrome Disease Activity Index
ESSPRI: European League Against Rheumatism Sjögren’s Syndrome Patient Reported Index
IFN: Interferon
Ig: Immunoglobulin
IQR: Interquartile range
LSG: Labial salivary gland
pSS: Primary Sjögren’s syndrome
SLE: Systemic lupus erythematosus
VAS: Visual analogue scale
W: Week
